# The factor structure of complex posttraumatic stress disorder in traumatized refugees

**DOI:** 10.3402/ejpt.v7.33253

**Published:** 2016-12-16

**Authors:** Angela Nickerson, Marylene Cloitre, Richard A Bryant, Ulrich Schnyder, Naser Morina, Matthis Schick

**Affiliations:** 1 School of Psychology, UNSW Australia, Sydney, NSW, Australia; 2 National Center for PTSD Dissemination and Training Division, Palo Alto, California, USA; 3 Department of Psychiatry and Psychotherapy, Zurich University Hospital, Zurich, Switzerland

**Keywords:** Refugees, torture, posttraumatic stress disorder, PTSD

## Abstract

**Background:**

The construct of complex posttraumatic stress disorder (CPTSD) has attracted much research attention in previous years, however it has not been systematically evaluated in individuals exposed to persecution and displacement. Given that CPTSD has been proposed as a diagnostic category in the ICD-11, it is important that it be examined in refugee groups.

**Objective:**

In the current study, we proposed to test, for the first time, the factor structure of CPTSD proposed for the ICD-11 in a sample of resettled treatment-seeking refugees.

**Method:**

The study sample consisted of 134 traumatized refugees from a variety of countries of origin, with approximately 93% of the sample having been exposed to torture. We used confirmatory factor analysis to examine the factor structure of CPTSD in this sample and examined the sensitivity, specificity, positive predictive power and negative predictive power of individual items in relation to the CPTSD diagnosis.

**Results:**

Findings revealed that a two-factor higher-order model of CPTSD comprising PTSD and Difficulties in Self-Organization (χ^2^ (47)=57.322, *p*=0.144, RMSEA=0.041, CFI=0.981, TLI=0.974) evidenced superior fit compared to a one-factor higher-order model of CPTSD (χ^2^ (48)=65.745, *p*=0.045, RMSEA=0.053, CFI=0.968, TLI=0.956). Overall, items evidenced strong sensitivity and negative predictive power, moderate positive predictive power, and poor specificity.

**Conclusions:**

Findings provide preliminary evidence for the validity of the CPTSD construct with highly traumatized treatment-seeking refugees.

**Highlights of the article:**

- Complex PTSD (CPTSD), proposed as a sibling diagnosis to posttraumatic stress disorder (PTSD) in ICD-11, has not been previously evaluated with treatment-seeking refugees.
- This study compared higher-order one and two-factor models of CPTSD using confirmatory factor analysis in a sample of traumatized refugees.
- Findings indicated that a higher-order two-factor model comprising PTSD and Disturbances in Self-Organization best fit the data.
- These findings provide preliminary evidence that the CPTSD construct is relevant to individuals exposed to persecution and displacement.

The World Health Organization has proposed substantial changes to the diagnostic category of “disorders associated with stress” in the 11th revision of the International Classification of Diseases and Related Health Problems (ICD-11). The Working Group for Trauma- and Stress-Related Disorders has suggested “sibling” diagnoses of posttraumatic stress disorder (PTSD) and the newly added complex PTSD (CPTSD) (Maercker et al., 2013b). Both of these require the individual to have been exposed to a traumatic event, from which he or she can be diagnosed with either PTSD or CPTSD. PTSD is diagnosed when one of two possible symptoms is exhibited in each of the following categories: reexperiencing of the traumatic event, avoidance of internal or external reminders of the event, and hyperarousal. Accordingly, PTSD symptoms are specifically related to the traumatic event and represent a predominantly fear-based response (Hyland et al., 2016). CPTSD is diagnosed when, in addition to meeting criteria for PTSD, trauma survivors exhibit disturbances in self-organization (DSO) by endorsing at least one of two possible symptoms in the categories of affective regulation, self-concept and interpersonal relations (Cloitre, Garvert, Brewin, Bryant, & Maercker, 2013; Maercker et al., 2013a, 2013b). In contrast to PTSD symptoms, DSO symptoms represent more pervasive changes in functioning across contexts (Hyland et al., 2016). While CPTSD has been proposed as a diagnostic category for ICD-11, considerable debate continues regarding the distinctiveness of the construct of CPTSD. CPTSD was excluded from the *Diagnostic and Statistical Manual*, 5th edition, following the argument of some commentators that the symptoms of CPTSD can be accommodated within the framework of existing definitions of PTSD (Resick et al., 2012). This assertion stems from the expansion of the diagnosis of PTSD in the DSM-5 to encompass symptoms such as self-blame, negative beliefs about the self and feeling alienated from others (American Psychiatric Association, 2013). The breadth of the DSM-5 PTSD diagnosis (Galatzer-Levy & Bryant, 2013) and the heterogeneity in potential clinical presentation afforded by these criteria (Cloitre, 2016) is in contrast to the ICD-11 constructs of PTSD and CPTSD which propose only six symptoms for the diagnosis of PTSD, with an additional six symptoms to meet criteria for CPTSD. Accordingly, the ICD-11 criteria may have benefits in terms of greater parsimony and usability in low-resource settings and reduced overlap with existing diagnoses (i.e., mood and anxiety disorders) (Maercker et al., 2013a), but still facilitates the distinction of symptom profiles that have been demonstrated to be readily observable by clinicians across cultural groups (Keeley et al., 2016).

CPTSD is considered to be especially likely to occur following exposure to repeated, prolonged, interpersonal trauma exposure. Consistent with this, there is growing evidence from research in western settings supporting the validity of this disorder with individuals exposed to sustained interpersonal trauma (Perkonigg et al., 2015), institutional abuse (Knefel, Garvert, Cloitre, & Lueger-Schuster, 2015^2015^), childhood abuse (Cloitre, Garvert, Weiss, Carlson, & Bryant, 2014), and people seeking treatment following exposure to a range of trauma types (Cloitre et al., 2013). While CPTSD was originally formulated to describe distinctive psychological responses arising from events where an individual is under the sustained and coercive control of a perpetrator (i.e., torture) (Herman, 1992), there has been scarce examination of CPTSD in individuals from non-western countries who have been exposed to persecution, mass trauma, and torture. It has been suggested that CPTSD and related constructs may be particularly relevant to these groups given the repeated and prolonged interpersonal trauma to which they are typically exposed (de Jong, Komproe, Spinazzola, van der Kolk, & van Ommeren, 2005; Morina & Ford, 2008; Palic & Elklit, 2014). In addition, refugees are displaced to unfamiliar environments, and may be unable to access important sources of support or established strategies for managing distress (e.g., work, leisure activities). These experiences may have an especially strong impact on the CPTSD domains of affect regulation, interpersonal relations and self-concept. Accordingly, it is important to investigate the construct of CPTSD in refugee groups. To our knowledge, the only study conducted to date investigating CPTSD in refugees was undertaken by Tay and colleagues (2015), who evaluated the factor structure of PTSD and CPTSD in West Papuan refugees displaced to Papua New Guinea. They found that the ICD-11 PTSD factor structure fit the data well, while the CPTSD factor structure did not, leading the authors to question the appropriateness of the CPTSD construct for trauma-exposed refugees. In this study, however, the authors tested the CPTSD construct as a single-factor higher-order model, with CPTSD being represented directly by six lower-order factors, namely, re-experiencing, avoidance, arousal, affective dysregulation, disturbances in self-concept, and disruptions in interpersonal relations. In contrast, CPTSD may be better conceptualized by a two-factor higher-order solution, where the two higher-order factors comprise PTSD and DSO, and each of these is represented by three lower-order factors (PTSD: re-experiencing, avoidance, and arousal; DSO: affective dysregulation, disturbances in self-concept, and disruptions in interpersonal relations). The authors of this article did not test this solution, and thus its validity has not yet been examined.

Three studies relevant to our investigation have been conducted examining the factor structure of the CPTSD construct in other trauma-affected samples. In addition to investigating a two-factor higher-order solution as described above, Hyland and colleagues (2016) examined (a) a single-factor model where all PTSD and CPTSD symptoms were indicators of a CPTSD factor, (b) a six-factor model of CPTSD whereby symptoms loaded onto the re-experiencing, avoidance, sense of threat (arousal), affective dysregulation, negative self-concept and disturbed relationships factors, and (c) a two-factor model in which PTSD symptoms loaded directly onto a PTSD factor and disturbances in self-organization symptoms loaded onto a DSO factor. PTSD symptoms were measured using six items from the Harvard Trauma Questionnaire (Mollica et al., 1992b), while DSO symptoms were measured using one item from the HTQ and five items from the Trauma Symptoms Checklist (Briere & Runtaz, 1989). This study found that the two-factor higher-order model best fit the data in a sample of childhood sexual abuse survivors. Karatzias and colleagues (2016) tested the factor structure of CPTSD with a heterogeneous sample of trauma survivors, using the newly developed ICD-11 Trauma Questionnaire to index PTSD and DSO symptoms. In addition to evaluating the factor structures specified by Hyland and colleagues (as described above), this study also tested a single higher-order factor structure, a model with two first-order factors and models where there was a hierarchical structure for the DSO but not PTSD items and where there was a hierarchical structure for the PTSD but not DSO items. Findings revealed that the model with two correlated higher-order factors yielded the best combination of model fit and parsimony. A third study conducted by Cloitre and colleagues (2013) tested a four-factor model of CPTSD, encompassing factors representing PTSD symptoms (which comprised three lower-order factors, namely re-experiencing, avoidance, and a sense of threat), affect dysregulation, negative self-concept, and interpersonal problems. They found good model fit for this solution; however, this model does not precisely match the current conceptualization of ICD-11 PTSD/CPTSD.

In this study, we proposed to conduct a preliminary evaluation of the ICD-11 factor structure of CPTSD in a sample of treatment-seeking refugees resettled in Switzerland using archival data. PTSD symptoms were measured using the Posttraumatic Diagnostic Scale (PDS; Foa, 1996; Foa, Cashman, Jaycox, & Perry, 1997), while DSO symptoms were drawn from the Difficulties in Emotion Regulation Scale (Gratz & Roemer, 2004), Experiences in Close Relationships measure (Wei, Russell, Mallinckrodt, & Vogel, 2007) and the Hopkins Symptom Checklist (Mollica, Wyshak, de Marneffe, Khuon, & Lavelle, 1987). Items were selected for inclusion in this study according to how closely they mapped onto the specific criteria proposed for the ICD-11. We also examined the psychometric properties of individual symptoms to determine their sensitivity, specificity, positive predictive power (PPV) and negative predictive power (NPV) in relation to the CPTSD diagnosis. An outstanding question is the extent to which PTSD and DSO represent distinctive but related constructs subsumed under the broader category of CPTSD. Accordingly, we compared the fit of (1) a single-factor higher-order model of CPTSD, which was represented by six lower-order factors (re-experiencing, avoidance, arousal, affective dysregulation, disturbances in self-concept, and disrupted interpersonal relations), and (2) a two-factor higher-order model of CPTSD comprising PTSD and DSO, with each of the higher-order factors being represented by three lower-order factors (PTSD: reexperiencing, avoidance, and arousal; DSO: affective dysregulation, disturbances in self-concept, and disruptions in interpersonal relations). If the first solution fits the data better, this would suggest that re-experiencing, avoidance, arousal, affective dysregulation, disturbances in self-concept, and disrupted interpersonal relations are best encompassed in a single overarching latent factor. This would provide evidence for the assertion that PTSD and DSO are not distinctive constructs. If the second solution fits the data better, this would suggest that PTSD and DSO are distinct but related constructs and provide evidence for the proposed ICD-11 diagnostic category of CPTSD with refugees. Our rationale for testing these models is that they allow us to determine the extent to which DSO symptoms are distinct but interrelated, reflecting the proposed conceptual organization of CPTSD for the ICD-11 (Maercker et al., 2013a). A recent survey conducted by Keely and colleagues indicated that clinicians' diagnostic accuracy is high in relation to the proposed ICD-11 criteria for PTSD and CPTSD, with low rates of miscategorization between the two disorders (Keeley et al., 2016). This combined with the finding from Hyland and colleagues (Hyland et al., 2016) that a higher-order two-factor solution was optimal in their investigation of PTSD and CPTSD in childhood sexual abuse survivors leads us to hypothesize that the two-factor higher-order solution will yield the best model fit.

## Method

### Participants

Participants in this study were 134 refugees and asylum-seekers who were receiving psychological treatment for trauma-related mental health problems at an outpatient unit for victims of torture and war in either Zurich or Bern, Switzerland. Inclusion criteria for participation in the study comprised (1) participants must be aged 18 or older, and (2) participants must speak one of the study languages (German, English, Turkish, Arabic, Farsi, and Tamil). Exclusion criteria encompassed (1) inability to use the tablet-based software used to collect data or complete self-report questionnaires, (2) pregnancy, (3) severe dissociative symptoms, (4) active psychosis, and (5) suicidality. No participants were excluded from this study. Of 152 patients considered eligible for the study and invited to participate, 137 provided informed consent (90.1%). Of these, three failed to attend the research session, thereby leading to a final sample size of 134 participants. The study was approved by the Ethics Committee of the Cantons of Zurich and Bern, Switzerland, and has therefore been performed in accordance with ethical standards laid down in the 1964 Declaration of Helsinki and its later amendments.

### Measures

Measures used in this study were translated into study languages by accredited translators. Gold-standard blind back-translation procedures also implemented (Bontempo, 1993), and differences rectified by independent bilingual translators who had experience in working with mental health constructs.

#### Trauma exposure

We assessed trauma exposure using a 23-item instrument developed for the current study. This scale represented the compilation of trauma event lists from two standardized questionnaires, namely the Harvard Trauma Questionnaire (HTQ) (Mollica et al., 1992) and the Posttraumatic Diagnostic Scale (PDS) (Foa, 1996; Foa, Cashman, Jaycox, & Perry, 1997). This scale indexed exposure to traumatic events commonly experienced by refugees, including witnessing the murder of loved ones, torture, deprivation of food, water, shelter, etc. We computed a total count of the number of traumatic events experienced by each participant, ranging from 0 to 23.

#### PTSD

We assessed symptoms of PTSD using the symptom subscale of the PDS (Foa, 1996; Foa et al., 1997). For this study, six items were extracted from the PDS as indicators for ICD-11 PTSD symptom clusters, including reexperiencing (PDS1: *Recurrent and intrusive distressing recollections of the event, including images, thoughts, or perceptions* and PDS2: *Recurrent distressing dreams of the event*), avoidance (PDS6: *Avoid thoughts, feelings, or conversations associated with the trauma* and PDS7: *Avoid activities, people, or places that remind you of the traumatic event*), and hyperarousal (PDS16: *Hypervigilance* and PDS17: *Exaggerated startle response*). Items were rated on a four-point scale (0=*not at all/only one time*, 1=*once a week or less/once in a while*, 2=*two to four times a week/half the time*, 3=*five or more times a week/almost always*). To determine a probable diagnosis and examine psychometric properties of the items, we also dichotomized symptoms as “present” or “absent”. Specifically, the symptom was coded as present if the participants rated it at the level of 2 or 3. Participants were considered to have a probable diagnosis of PTSD if they reported at least one symptom from each of the reexperiencing, avoidance, and arousal clusters, and did not meet criteria for CPTSD.

#### CPTSD

We assessed symptoms of CPTSD using six items derived from scales implemented in this study. Participants were considered to have a probable diagnosis of CPTSD, if in addition to meeting the criteria for PTSD, they reported DSO where they endorsed at least one symptom from each of the affect dysregulation, disturbances in self-concept, and difficulties in interpersonal relationships domains.

#### Affect dysregulation

Affect dysregulation was measured using two items from the Difficulties in Emotion Regulation Scale (DERS) (Gratz & Roemer, 2004), namely, *When I'm upset, I believe that I will remain that way for a long time* (DERS10) and *When I'm upset, I have difficulty controlling my behaviors* (DERS16). Items in the DERS are rated on a five-point scale (1=*almost never*, 2=*sometimes*, 3=*about half the time*, 4=*most of the time*, 5=*almost always*). Items scored as 3, 4, or 5 were coded as present.

#### Disturbances in self-concept

Disturbances in self-concept was measured using two items from the Hopkins Symptom Checklist (Mollica, Wyshak, De Marneffe, Khuon, & Lavelle, 1987), a measure of depression and anxiety that is widely implemented with refugees (Carlsson, Mortensen, & Kastrup, 2005; Schweitzer, Melville, Steel, & Lacharez, 2006). These items included *Blaming yourself for things* (HSCL2) and *Feelings of worthlessness* (HSCL15). Items on the HSCL were rated on a four-point scale (1=*not at all*, 2=*a little*, 3=*quite a bit*, 4=*extremely*). Items rated as 3 or 4 were coded as being present.

#### Difficulties in interpersonal relations

Items indexing difficulties in interpersonal relations were derived from the Experiences in Close Relationships Scale (ECR) and the PDS. The ECR (Wei, Russell, Mallinckrodt, & Vogel, 2007) is a measure of adult attachment style. The item extracted from this scale was *I avoid getting too close to others* (ECR9). This item was rated on a seven-point scale (1=*not at all*, 2=*a little bit*, 3=*less than moderately*, 4=*moderately*, 5=*more than moderately*, 6=*a lot*, 7=*extremely*). Items scored as 4, 5, 6, or 7 were coded as present. One item was also extracted from the PDS scale to represent the difficulties in interpersonal relations cluster of CPTSD (PDS10: *Feelings of detachment or estrangement from others*).

### Procedure

Upon attending the study session, participants first completed written informed consent. Measures were implemented using a therapist-assisted computer-based assessment tool (Knaevelsrud & Müller, 2007), which participants used to read and/or listen to each item and the range of possible responses in their own language. Participants then indicated their response by touching the screen. The research assessment lasted 60–120 min, with participants being assisted either by a psychiatrist or clinical psychologist with a minimum of 3 years’ experience in working with refugee groups or by a supervised masters-level student of clinical psychology who had received extensive training. Participants were reimbursed with CHF40 (approximately US$40) for participation.

### Data analysis

Confirmatory factor analysis (CFA) was used to evaluate the fit of the proposed ICD-11 CPTSD factor structure. Analyses were conducted using Mplus Version 7.4 (Muthén & Muthén, 1998–2010), with a maximum likelihood estimator. The following indices were used to evaluate model fit: Root Mean Square Error of Approximation (RMSEA) <0.06, and Comparative Fit Index (CFI) and Tucker–Lewis index (TLI) values approaching 0.95 or greater (Hu & Bentler, 1998). There was less than 5% missing data on any variable. Data on all variables was normally distributed. Missing data was accounted for using maximum likelihood estimation.

We first tested a two-factor higher-order model of CPTSD, which reflects the factor structure proposed for ICD-11. This model encompasses two second-order factors, namely PTSD and DSO, with each comprising three first-order factors that were derived from two indicator variables for each factor. For PTSD, the three first-order factors included reexperiencing symptoms, with indicators comprising *Recurrent and intrusive distressing recollections of the event, including images, thoughts, or perceptions* (PDS1) and *Recurrent distressing dreams of the event* (PDS2); avoidance symptoms, with indicators comprising *Avoid thoughts, feelings, or conversations associated with the trauma* (PDS6) and *Avoid activities, people, or places that remind you of the traumatic event* (PDS7), and arousal symptoms, with indicators comprising *Hypervigilance* (PDS16) and *Exaggerated startle response* (PDS17).

For DSO, the three first-order factors were negative self-concept, with indicators constituting *Blaming yourself for things* (HSCL2) and *Feelings of worthlessness* (HSCL15); interpersonal problems, with indicators constituting *Feelings of detachment or estrangement from others* (PDS10) and *I avoid getting too close to others* (ECR9); and emotion dysregulation, with indicators including *When I'm upset, I believe that I will remain that way for a long time* (DERS10) and *When I'm upset, I have difficulty controlling my behaviors* (DERS16).

Next we tested a one-factor higher-order model, in which the single higher-order factor CPTSD was comprised of the six first-order factors described above. Finally, we calculated the sensitivity, specificity, PPV, and NPV for each of the symptoms in relation to the CPTSD diagnosis as proposed for the ICD-11. Sensitivity was defined as the probability of the presence of the symptom when the diagnosis is present, specificity was defined as the probability of the absence of the symptom when the diagnosis is absent, PPV was defined as the probability that the disorder is present when the symptom is present, and NPV was defined as the probability that the diagnosis is absent when the symptom is absent.

## Results

### Participant characteristics

Participants in this study had a mean age of 42.4 years (SD=9.8), with approximately three-quarters of the sample being male (*N*=105, 78.4%). Participants were from a variety of countries of origin, including Turkey (*N*=71, 53%, with *N*=58, 43.3% being Kurdish), Iran (*N*=15, 12%), Sri Lanka (*N*=11, 8%), Bosnia (*N*=6, 5%), Iraq (*N*=6, 5%), Afghanistan (*N*=5, 4%), and others (*N*=20, 13%). Participants had been exposed to a mean of 13.11 (SD=4.80) types of traumatic events, with over 90% of the sample having experienced torture (*N*=114, 92.7%). Frequency of exposure to specific trauma types is presented in Table 1. Participants had lived in Switzerland for a mean of 9.01 years (SD=6.67). Approximately one-fifth of the sample (*N*=70, 19.7%) had a probable diagnosis of PTSD according to the ICD-11 criteria, while one-third of the sample (*N*=44, 32.8%) met criteria for CPTSD. Correlations between CPTSD and PTSD sub-scales are presented in Table 2.

### Confirmatory factor analysis

**Fig. 1:**
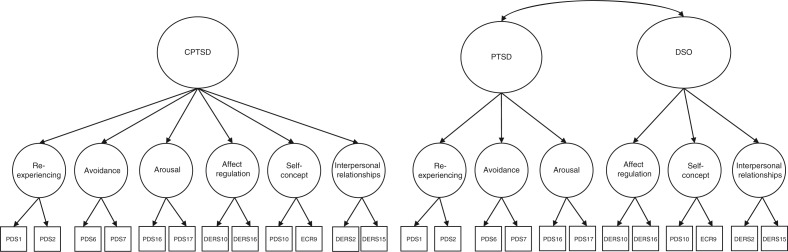
One- and two-factor higher-order models of CPTSD.

**Fig. 2:**
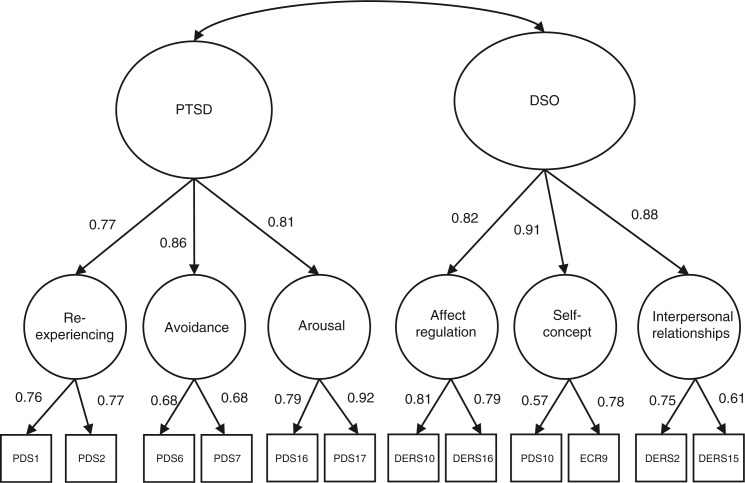
Two-factor higher-order model of CPTSD (standardized factor loadings).

The two models tested in this study are presented in Fig. 1 and model fit is presented in Table 3. In the two-factor model, the correlation between PTSD and CPTSD was 0.84 (*p<*0.001). While the one-factor higher-order model also fit the data well a smaller (non-significant) chi square value revealed that the two-factor model yielded better model fit. This was substantiated by a smaller RMSEA, larger CFI, TLI and smaller AIC and BIC. Factor loadings for each of the indicator variables and first-order factors are presented in Table 4 and Fig. 2. All standardized factor loadings were greater than 0.65, with the exception of *Blaming yourself for things*, which had a standardized factor loading of 0.57, and *Avoiding getting close to others*, which had a standardized factor loading of 0.61.

**Table 1:** Trauma exposure reported by refugees and asylum seekers

Trauma type	*n*	%
Torture	124	92.5
Enforced isolation from others	102	76.1
Imprisonment	99	73.9
Non-sexual assault by a stranger	95	71.0
Combat situation	94	70.1
Being close to death	91	67.9
Murder of a family member or friend	84	62.7
Unnatural death of a family member or friend	80	59.7
Lack of food or water	79	60.0
Forced separation from family member	75	56.0
Ill health without access to medical care	69	56.1
Serious physical injury	66	51.5
Lack of shelter	65	48.5
Disappearance or kidnapping	62	46.3
Brainwashing	62	46.3
Non-sexual assault by a family member or someone you know	56	41.8
Serious accident, fire or explosion	52	38.8
Murder of one or more strangers	46	34.3
Natural disaster	45	33.6
Sexual assault by a stranger	44	32.8
Life-threatening illness	32	23.9
Sexual contact when you were younger than 18 with someone who was 5 or more years older than you	23	17.2
Sexual assault by a family member or someone you know	19	14.2

**Table 2:** Correlations between PTSD and DSO subscales

		1	2	3	4	5
1	PTSD – Re-experiencing					
2	PTSD – Avoidance	0.48**				
3	PTSD – Arousal	0.48**	0.49**			
4	DSO – Affect Dysregulation	0.37**	0.40**	0.48**		
5	DSO – Disturbances in Self-Concept	0.36**	0.45**	0.46**	0.50**	
6	DSO – Difficulties in Interpersonal Relationships	0.27**	0.24**	0.30**	0.47**	0.21*

* *p*<0.01,

** *p*<0.001

**Table 3:** Model fit statistics for one-factor and two-factor higher order models

	χ^2^	df	*p*	RMSEA	CFI	TLI	AIC	SS-BIC
One-factor model	65.75	48	0.045	0.053	0.968	0.956	4430.755	4419.608
Two-factor model	57.32	47	0.1444	0.040	0.981	0.974	4424.332	4412.920

**Table 4:** Factor loadings of indicator variables and first-order factors in one-factor higher order model and two-factor higher-order model

Symptom	One-factor model	Two-factor model
PTSD symptoms		
Re-experiencing	1.00 (0.72)	1.00 (0.77)
Recurrent and intrusive distressing recollections of the event, including images, thoughts, or perceptions (PDS1)	1.00 (0.77)	1.00 (0.76)
Recurrent distressing dreams of the event (PDS2)	1.07 (0.76)	1.10 (0.77)
Avoidance	1.17 (0.82)	1.16 (0.86)
Avoid thoughts, feelings, or conversations associated with the trauma (PDS6)	1.00 (0.69)	1.00 (0.68)
Avoid activities, people or places that remind you of the traumatic event (PDS7)	1.01 (0.67)	1.10 (0.68)
Arousal	1.31 (0.77)	1.31 (0.81)
Hyper-vigilance (PDS16)	1.00 (0.79)	1.00 (0.79)
Exaggerated startle response (PDS17)	1.09 (0.92)	0.98 (0.92)
Disturbances in self-organisation		
Affect dysregulation	1.65 (0.78)	1.00 (0.82)
When I'm upset, I believe that I will remain that way for a long time (DERS10)	1.00 (0.83)	1.00 (0.81)
When I'm upset, I have difficulty controlling my behaviours (DERS16)	0.95 (0.78)	1.53 (0.79)
Negative self-concept	1.02 (0.86)	0.62 (0.91)
Blaming yourself for things (PDS10)	1.00 (0.57)	1.00 (0.57)
Feelings of worthlessness (ECR9)	1.40 (0.77)	1.41 (0.78)
Interpersonal problems	1.42 (0.86)	0.83 (0.88)
Feelings of detachment or estrangement from others (DERS2)	1.00 (0.76)	1.00 (0.75)
I avoid getting too close to others (DERS15)	1.49 (0.60)	1.53 (0.61)

Unstandardized factor loading (standardized factor loading).

**Table 5:** Frequency, sensitivity, specificity, positive predictive power, and negative predictive power of ICD PTSD and disturbances in self-organization symptoms in relation to ICD C-PTSD diagnosis

Symptom	Frequency N (%)	Sensitivity	Specificity	Positive predictive power	Negative predictive power
PTSD symptoms					
Re-experiencing symptoms					
Recurrent and intrusive distressing recollections of the event, including images, thoughts, or perceptions (PDS1)	99 (73.9%)	0.98	0.36	0.42	0.97
Recurrent distressing dreams of the event (PDS2)	88 (65.7%)	0.96	0.49	0.48	0.96
Avoidance					
Avoid thoughts, feelings, or conversations associated with the trauma (PDS6)	82 (61.2%)	0.93	0.54	0.50	0.94
Avoid activities, people, or places that remind you of the traumatic event (PDS7)	73 (54.5%)	0.91	0.60	0.53	0.93
Arousal					
Hypervigilance (PDS16)	76 (56.7%)	0.93	0.58	0.53	0.94
Exaggerated startle response (PDS17)	81 (60.4%)	0.98	0.55	0.52	0.98
Disturbances in self-organization					
Affect dysregulation					
When I'm upset, I believe that I will remain that way for a long time (DERS10)	65 (48.5%)	0.89	0.71	0.60	0.93
When I'm upset, I have difficulty controlling my behaviors (DERS16)	58 (43.3%)	0.77	0.73	0.59	0.87
Negative self-concept					
Blaming yourself for things (PDS10)	77 (57.5%)	0.81	0.53	0.46	0.86
Feelings of worthlessness (ECR9)	79 (59.0%)	0.89	0.56	0.49	0.91
Interpersonal problems					
Feelings of detachment or estrangement from others (DERS2)	79 (59.0%)	0.93	0.57	0.52	0.94
I avoid getting too close to others (DERS15)	48 (35.8%)	0.67	0.78	0.60	0.83

PTSD, posttraumatic stress disorder; CPTSD, complex posttraumatic stress disorder. Sensitivity: The probability of the presence of the symptom when PTSD or C-PTSD diagnosis is present. Specificity: The probability of the absence of the symptom when PTSD or C-PTSD diagnosis is absent. Positive predictive power: The probability of the presence of PTSD or C-PTSD diagnosis when the symptom is present. Negative predictive power: The probability of the absence of PTSD or C-PTSD diagnosis when the symptom is absent.

### Sensitivity, specificity, positive PPV and NPV of symptoms in relation to CPTSD diagnoses

Results (presented in Table 5) indicated that virtually all items demonstrated good sensitivity (≥0.75), with the exception of the item *I avoid getting too close to others* (sensitivity=0.67). In contrast, items demonstrated relatively weaker specificity. In particular, the reexperiencing items had poor specificity (*Recurrent and intrusive distressing recollections* specificity=0.36, *Recurrent distressing dreams* specificity=0.49). This suggests that many individuals who did have re-experiencing symptoms did not meet criteria for CPTSD. In contrast, items relating to affect dysregulation evidenced fairly strong specificity (*When I'm upset, I believe that I will remain this way for a long time* specificity=0.71, *When I'm upset, I have difficulty controlling my behaviors* specificity=0.73). Similarly, one of the items relating to interpersonal problems had strong specificity (*I avoid getting too close to others* specificity=0.78), although the other item evidenced relatively poor specificity (*Feelings of detachment or estrangement from others* specificity=0.57). Most items evidenced moderate PPV, with *recurrent and intrusive distressing recollections* being particularly weak (PPV=0.42). All items evidenced excellent NPV (NPV=0.83 to 0.98).

## Discussion

The current study conducted a preliminary evaluation of the factor structure for the proposed ICD-11 diagnosis for CPTSD using archival data in a sample of severely traumatized refugees, the majority of whom had been exposed to torture. Findings indicated that the two-factor higher-order solution evidenced the best model fit, providing support for the conceptualization of CPTSD as being two-dimensional, comprising PTSD symptoms and DSO. The finding that this model fits the data well adds to the growing evidence that CPTSD represents a valid construct and is in accordance with the research conducted with a variety of trauma-exposed groups (Cloitre et al., 2013, 2014; Knefel et al., 2015; Perkonigg et al., 2016). Further, these findings extend the current evidence base to support the applicability of the CPTSD construct to individuals exposed to persecution, torture, and displacement.

Results from the current study are broadly consistent with the findings of Hyland and colleagues (2016) and Karatzias and colleagues (2016) that a two-factor higher-order model best fit the data in a sample of survivors of childhood sexual abuse. In contrast, Tay and colleagues (2015) found poor model fit in a factor analysis examining CPTSD in a sample of West Papuan refugees. In this study, however, CPTSD was conceptualized as a unidimensional construct, with PTSD symptom clusters (comprising re-experiencing, avoidance, and hyperarousal), affect dysregulation, difficulties in interpersonal relationships, and self-concept disturbances all loading onto a single CPTSD factor. In contrast, we evaluated CPTSD as a two-factor construct – comprising PTSD and DSO – reflecting the conceptualization of CPTSD as a sibling disorder to PTSD, which incorporates an additional (and distinct) set of symptom clusters relating to affect regulation, interpersonal relationships, and self-concept. Results from the current study indicated that representing PTSD and DSO as separate (but correlated) factors resulted in better model fit. As Tay et al. (2015) did not test this model, it is not possible to know whether a bi-dimensional representation of CPTSD would also evidence relatively better model fit with their sample. It may also be the case that CPTSD as a construct may have particular relevance to treatment-seeking refugees resettled in a western setting as opposed to individuals displaced in lower and middle-income countries who continue to face substantial ongoing threat. For example, CPTSD may be especially salient amongst treatment-seeking refugees with high levels of psychopathology. Alternatively, CPTSD may represent a less cohesive construct in the context of ongoing threat such as that often experienced by refugees displaced within the developing world, where fear-based symptoms such as those that from part of the PTSD constellation may dominate clinical presentations. Further research is required to determine the generalizability of CPTSD beyond the current sample of resettled refugees.

The findings from the current study provide preliminary evidence for the distinct but related nature of the PTSD and DSO constructs in a refugee sample, supporting the conceptualization of PTSD and CPTSD as sibling diagnoses in the ICD-11. It is notable that the two-factor model tested in the current study yielded strong standardized factor loadings in both the higher- and lower-order factors. In particular, the higher-order factor loadings for the symptom clusters subsumed under DSO evidenced strong loadings (greater than 0.80), suggesting that these clusters were good indicators of the DSO construct and were strongly related to one another. Overall, these results further support the validity of these symptoms as indicators of the CPTSD construct.

Examination of the sensitivity, specificity, PPV, and NPV of items in relation to the CPTSD diagnosis indicated that there was considerable variation in item performances. Items generally performed better in terms of sensitivity and NPV than specificity and PPV. Notably, reexperiencing symptoms including intrusive memories and nightmares evidenced low specificity and PPV in the current sample, indicating that the absence of these symptoms was not well able to predict the absence of the CPTSD diagnosis or vice versa. This is consistent with findings from our previous investigation of DSM-5 PTSD symptoms in the same sample (Schnyder et al., 2015). In this study, the reexperiencing items evidenced relatively poor specificity (memories specificity=0.40, dreams specificity=0.50) and PPV (memories specificity=0.60, dreams specificity=0.61) in relation to the DSM-5 PTSD diagnosis (Schnyder et al., 2015). Considering that intrusive memories were reported by nearly three-quarters of the sample, and nightmares by two-thirds of the sample, it may be that these symptoms are not well able to discriminate between those with and without trauma-related disorders, regardless of whether it is CPTSD or PTSD.

With regard to the DSO items, those indexing affect dysregulation yielded the best balance of sensitivity/specificity/PPV/NPV. This is consistent with findings from the same sample that emotion regulation difficulties are associated with trauma exposure and post-migration stressors, and are related to psychopathology (Nickerson et al., 2015).The negative self-concept items demonstrated strong sensitivity and PPV, but relatively weak specificity and NPV, suggesting that while many individuals with CPTSD reported these symptoms, so did many without CPTSD. The negative self-concept items demonstrated strong sensitivity and PPV, but relatively weak specificity and NPV, suggesting that while many individuals with CPTSD reported these symptoms, so did many without CPTSD. It may be the case that changes in self-concept arose from the specific experiences of the sample rather than being associated specifically with CPTSD. Notably, over 90% of the sample had been tortured, an experience which has been consistently linked to changes in identity by clinicians and researchers (Nickerson, Bryant, Rosebrock, & Litz, 2014). Further research is required to determine how aspects of the refugee experience interact with specific symptoms and diagnosis. Interestingly, the two items relating to interpersonal problems evidenced different patterns of sensitivity and specificity. While feelings of detachment demonstrated relatively higher specificity (0.93) than sensitivity (0.57), this pattern was reversed (and weaker) in the item relating to avoidance of getting too close to others (sensitivity=0.67, specificity=0.78). These items may be tapping into different types of interpersonal problems; while feelings of detachment (which a symptom of PTSD in the DSM-5 criteria for the disorder) may represent an internal experience, avoidance of becoming close to others may indicate a more active strategy that leads to interpersonal dysfunction. Accordingly, the former symptom was reported more frequently than the latter (59% vs. 36%). The experience of avoiding getting too close to others may also be particularly subject to cultural influence and may have a different meaning in collectivist societies (from which the majority of participants in this study were drawn) compared to individualist societies. Further research investigating these individual symptoms among individuals from diverse cultural backgrounds is required to determine which items yield the optimal balance of psychometric properties.

It is notable that rates of probable PTSD (19.7%) and CPTSD (32.8%) were high in the current sample. Meta-analytic findings suggest that the prevalence of PTSD in refugee groups is approximately 30% (Steel et al., 2009). As the proposed classification system for PTSD and CPTSD specifies that individuals cannot meet criteria for both disorders, rates of PTSD were substantially lower in this study as individuals who would have otherwise met criteria for probable PTSD were subsumed into the CPTSD diagnosis. While there has been scant investigation of CPTSD amongst refugees, the number of individuals who met criteria for this diagnosis in the current study is much higher than that in the research conducted by Tay and colleagues (2015), where only 3% met criteria for CPTSD (and 6% for PTSD). Another study conducted with trauma-affected young adults in Uganda (Murphy et al., 2016) found that 34% of the sample met the ICD-11 criteria for PTSD and 21% met the criteria for CPTSD. The high rates of CPTSD in our study is likely due to the treatment-seeking nature of our sample. Accordingly, rates of CPTSD in our study are consistent with other treatment-seeking samples. For example, in a study conducted with treatment-seeking childhood abuse survivors (Cloitre et al., 2014), 38% of the sample had a probable CPTSD diagnosis.

From a clinical perspective, the finding that CPTSD symptoms were relatively highly endorsed in the current sample highlights the potential limitations of current best-practice treatment approaches for PTSD, which focus primarily on the reduction of fear-related symptoms (Foa, Keane, Friedman, & Cohen, 2008). It may be the case that these interventions are less effective in addressing symptoms comprising DSO, for example, disruptions in interpersonal relationships or self-concept. There is emerging evidence supporting the use of interventions, such as Skills Training in Affective and Interpersonal Regulation, which aims to enhance clients’ emotion regulation capacity and interpersonal skills prior to engaging in traditional exposure-based interventions for PTSD (Cloitre, Koenen, Cohen, & Han, 2002; Cloitre et al., 2010). The utility of these phase-based interventions has accordingly been recognized in the Guidelines for the Treatment of Complex PTSD in Adults, developed by the International Society for Traumatic Stress Studies (Cloitre et al., 2012). While these interventions have not yet been tested in refugee samples, they may represent a promising direction of future enquiry to inform treatment of CPTSD in individuals exposed to human rights violations and displacement.

The current study had a number of limitations. First and foremost, items indexing DSO symptoms in the current study were derived from various scales instead of a validated measure of this construct, which may have affected participant responses. Nevertheless, a number of the studies investigating CPTSD to date have employed a similar strategy, using archival data or items from a variety of scales to examine these symptom constellations (Cloitre et al., 2013, 2014; Hyland et al., 2016; Perkonigg et al., 2016). Despite using different scales, our findings were consistent with those of Hyland and colleagues (2016) who reported that the higher-order two factor solution evidenced the best model fit in survivors of childhood sexual abuse. Further research investigating the structure of CPTSD using a standardized scale such as the ICD-11 Trauma Questionnaire (Cloitre, Roberts, Bisson, & Brewin, 2015), as in the study conducted by Karatzias and colleagues (2016), would strengthen conclusions regarding the factor structure of CPTSD. Second, all participants in this study were treatment-seeking, and it is not possible to know the extent to which these symptoms may have been impacted by ongoing therapy. For example, it may be the case that treatment had reduced PTSD symptoms while CPTSD symptoms were more resistant to intervention, which may have influenced the model fit. Third, while participants were from a variety of countries of origin, the vast majority of them had been exposed to torture, and thus it is not possible to determine whether these results are generalizable to refugee groups who were not exposed to torture; in addition, sample size precluded investigation of whether CPTSD differs across cultural groups. Fourth, we did not examine other diagnoses (e.g., depression and borderline personality disorder), and it would be useful in future studies to assess the discriminant validity of the CPTSD construct. Fifth, we did not assess for exposure to childhood trauma, and thus it is not possible to determine the extent to which symptoms of CPTSD or PTSD may have been attributable to these experiences.

To our knowledge, this is the first study to investigate the factor structure of CPTSD in a sample of tortured refugees resettled in a western country. Findings provide preliminary evidence for the utility and validity of the CPTSD construct in refugees from variety of cultural backgrounds who have been exposed to extreme human rights violations. This research adds to the growing body of evidence validating the CPTSD construct in trauma-affected populations. The finding that CPTSD is prevalent in traumatized refugees may point to the development and implementation of specific psychological interventions to ameliorate the devastating psychological impact of strategic human-instigated traumatic events such as torture.
